# 6-Bromo-1-methyl-4-[2-(4-methyl­benzyl­idene)hydrazinyl­idene]-3*H*-2λ^6^,1-benzothia­zine-2,2-dione

**DOI:** 10.1107/S1600536811028406

**Published:** 2011-07-23

**Authors:** Muhammad Shafiq, Islam Ullah Khan, Muhammad Zia-ur-Rehman, Muhammad Nadeem Arshad, Abdullah M. Asiri

**Affiliations:** a Materials Chemistry Laboratory, Department of Chemistry, GC University, Lahore 54000, Pakistan; b Applied Chemistry Research Center, PCSIR Laboratories Complex, Ferozpur Road, Lahore 54600, Pakistan; c X-ray Diffraction and Physical Laboratory, Department of Physics, School of Physical Sciences, University of the Punjab, Quaid-e-Azam Campus, Lahore 54590, Pakistan; d The Center of Excellence for Advanced Materials Research, King Abdul Aziz University, Jeddah, PO Box 80203, Saudi Arabia

## Abstract

In the title compound, C_17_H_16_BrN_3_O_2_S, the two fused rings are twisted by a dihedral angle of 6.61 (15)°. The thia­zine ring adopts a sofa conformation. The toluene ring is oriented at dihedral angles of 15.5 (2) and 20.6 (2)° with respect to the bromo­benzene and thia­zine rings, respectively. The benzyl­idene system is approximately planar [r.m.s. deviation = 0.0388 Å]. In the cyrstal, weak inter­molecular C—H⋯O hydrogen bonds connects the mol­ecules into a chain along the *b* axis.

## Related literature

For the synthesis of the title compound, see: Shafiq *et al.* (2011 ▶). For related structures, see: Khan *et al.* (2010 ▶); Shafiq *et al.* (2009 ▶); Arshad *et al.* (2009 ▶).
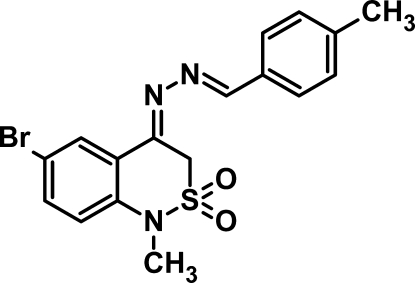


## Experimental

### 

#### Crystal data

C_17_H_16_BrN_3_O_2_S
*M* *_r_* = 406.30
Monoclinic, 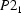

*a* = 9.1077 (6) Å
*b* = 6.8328 (4) Å
*c* = 14.1765 (9) Å
β = 96.807 (3)°
*V* = 876.00 (10) Å^3^
*Z* = 2
Mo *K*α radiation
μ = 2.48 mm^−1^
*T* = 296 K
0.32 × 0.12 × 0.10 mm

#### Data collection

Bruker Kappa APEXII CCD diffractometer
Absorption correction: multi-scan (*SADABS*; Bruker, 2007 ▶) *T*_min_ = 0.504, *T*_max_ = 0.790
10166 measured reflections
4119 independent reflections
2881 reflections with *I* > 2σ(*I*)
*R*_int_ = 0.026

#### Refinement

*R*[*F* ^2^ > 2σ(*F* ^2^)] = 0.034
*wR*(*F* ^2^) = 0.078
*S* = 0.97
4119 reflections
220 parameters
1 restraint
H-atom parameters constrained
Δρ_max_ = 0.29 e Å^−3^
Δρ_min_ = −0.36 e Å^−3^
Absolute structure: Flack (1983 ▶), 1771 Friedel pairs
Flack parameter: 0.004 (8)

### 

Data collection: *APEX2* (Bruker, 2007 ▶); cell refinement: *SAINT* (Bruker, 2007 ▶); data reduction: *SAINT*; program(s) used to solve structure: *SHELXS97* (Sheldrick, 2008 ▶); program(s) used to refine structure: *SHELXL97* (Sheldrick, 2008 ▶); molecular graphics: *PLATON* (Spek, 2009 ▶); software used to prepare material for publication: *WinGX* (Farrugia, 1999 ▶) and *PLATON*.

## Supplementary Material

Crystal structure: contains datablock(s) I, global. DOI: 10.1107/S1600536811028406/pv2429sup1.cif

Structure factors: contains datablock(s) I. DOI: 10.1107/S1600536811028406/pv2429Isup2.hkl

Supplementary material file. DOI: 10.1107/S1600536811028406/pv2429Isup3.cml

Additional supplementary materials:  crystallographic information; 3D view; checkCIF report

## Figures and Tables

**Table 1:** Hydrogen-bond geometry (Å, °)

*D*—H⋯*A*	*D*—H	H⋯*A*	*D*⋯*A*	*D*—H⋯*A*
C17—H17*C*⋯O1^i^	0.96	2.64	3.546 (5)	158

Symmetry code: (i) 
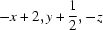
.

**Fig. 1.**
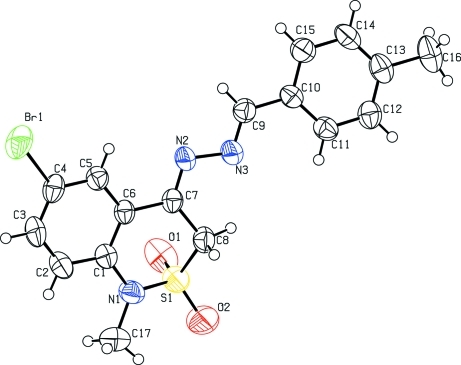
The ORTEP diagram of the title molecule showing the thermal ellipsoids drawn at 50% probability level.

**Fig. 2.**
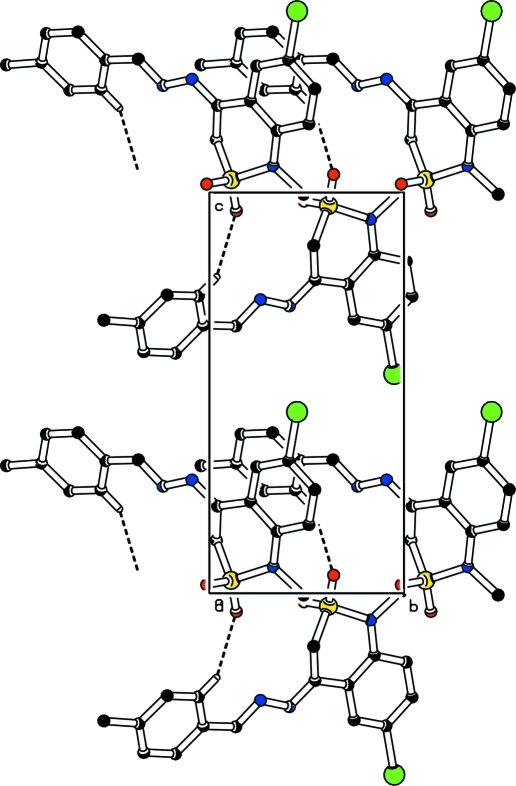
A unit cell diagram of the title compound showing C—H···O type interactions using dashed lines.

## supplementary crystallographic information

### Comment

In the title molecule (Fig. 1), the thiazine ring adopts a sofa conformation which is similar to the conformation of the corresponding ring in a previously reported compoud, 1-propyl-1*H*-2,1-benzothiazin-4(3*H*)-one 2,2-dioxide (Khan *et al.*, 2010). The aromatic ring (C1—C6) in the title compound is twisted at 15.5 (2)° with respect to the touluene ring (C10—C15). The benzylidene moiety (C9—C16/N1/N2) is also very near to planerity as showing the r. m. s. deviation of 0.0388Å and is oriented at a dihedral angle 15.02 (12)° with respect to the benzene ring (C1—C6). The fused aromatic (C1—C6) and thiazine (C1/C6/C7/C8/N1/S1) rings are twisted at a dihedral angle 6.61 (15)°. The oxygen atom from SO_2_ group is involved in weak C—H···O type hydrogen bonding interaction, which connects the molecules in a zig-zag mode along the *b*-axis.

The crystal structures of several molecules related to the title compound have been reported (Arshad *et al.*, 2009; Shafiq *et al.*, 2009).

### Experimental

The synthesis of the title compound has already been reportrd (Shafiq *et al.*, 2011). It was recrystalized from a solution in dry ethanol.

### Refinement

All H-atoms were positioned at idealized geometry with C_aromatic_—H = 0.93 Å and C_methyl_—H = 0.96 Å and were refined using a riding model with *U*_iso_(H) = 1.2 *U*_eq_(C) for aromatic & *U*_iso_(H) = 1.5 *U*_eq_(C) for methyl carbon atoms. The reflection 0 0 1 has been omitted in final refinement. An absolute structure was determined (Flack, 1983) using 1771 Friedel pairs.

### Crystal data

**Table:**

C_17_H_16_BrN_3_O_2_S	*F*(000) = 412
*M**_r_* = 406.30	*D*_x_ = 1.540 Mg m^−^^3^
Monoclinic, *P*2_1_	Mo *K*α radiation, λ = 0.71073 Å
Hall symbol: P 2yb	Cell parameters from 3801 reflections
*a* = 9.1077 (6) Å	θ = 2.3–23.7°
*b* = 6.8328 (4) Å	µ = 2.48 mm^−^^1^
*c* = 14.1765 (9) Å	*T* = 296 K
β = 96.807 (3)°	Needle, yellow
*V* = 876.00 (10) Å^3^	0.32 × 0.12 × 0.10 mm
*Z* = 2	

### Data collection

**Table:**

Bruker Kappa APEXII CCD diffractometer	4119 independent reflections
Radiation source: fine-focus sealed tube	2881 reflections with *I* > 2σ(*I*)
graphite	*R*_int_ = 0.026
φ and ω scans	θ_max_ = 28.3°, θ_min_ = 2.5°
Absorption correction: multi-scan (*SADABS*; Bruker, 2007)	*h* = −12→12
*T*_min_ = 0.504, *T*_max_ = 0.790	*k* = −8→9
10166 measured reflections	*l* = −18→18

### Refinement

**Table:**

Refinement on *F*^2^	Secondary atom site location: difference Fourier map
Least-squares matrix: full	Hydrogen site location: inferred from neighbouring sites
*R*[*F*^2^ > 2σ(*F*^2^)] = 0.034	H-atom parameters constrained
*wR*(*F*^2^) = 0.078	*w* = 1/[σ^2^(*F*_o_^2^) + (0.0256*P*)^2^] where *P* = (*F*_o_^2^ + 2*F*_c_^2^)/3
*S* = 0.97	(Δ/σ)_max_ = 0.018
4119 reflections	Δρ_max_ = 0.29 e Å^−^^3^
220 parameters	Δρ_min_ = −0.36 e Å^−^^3^
1 restraint	Absolute structure: Flack (1983), 1771 Friedel pairs
Primary atom site location: structure-invariant direct methods	Flack parameter: 0.004 (8)

### Special details

**Table:**

Geometry. All esds (except the esd in the dihedral angle between two l.s. planes) are estimated using the full covariance matrix. The cell esds are taken into account individually in the estimation of esds in distances, angles and torsion angles; correlations between esds in cell parameters are only used when they are defined by crystal symmetry. An approximate (isotropic) treatment of cell esds is used for estimating esds involving l.s. planes.
Refinement. Refinement of F^2^ against ALL reflections. The weighted R-factor wR and goodness of fit S are based on F^2^, conventional R-factors R are based on F, with F set to zero for negative F^2^. The threshold expression of F^2^ > 2sigma(F^2^) is used only for calculating R-factors(gt) etc. and is not relevant to the choice of reflections for refinement. R-factors based on F^2^ are statistically about twice as large as those based on F, and R- factors based on ALL data will be even larger.

### Fractional atomic coordinates and isotropic or equivalent isotropic displacement parameters (Å^2^)

**Table:**

	*x*	*y*	*z*	*U*_iso_*/*U*_eq_	
Br1	1.13358 (4)	1.44957 (7)	0.45270 (2)	0.08174 (14)	
S1	0.72100 (10)	1.11072 (10)	0.03233 (5)	0.0611 (2)	
O1	0.8404 (3)	0.9855 (3)	0.02125 (18)	0.0855 (8)	
O2	0.6089 (3)	1.1379 (5)	−0.04538 (16)	0.0999 (9)	
N1	0.7821 (3)	1.3277 (3)	0.06656 (16)	0.0589 (6)	
N2	0.7702 (3)	0.9100 (3)	0.28328 (17)	0.0519 (6)	
N3	0.6650 (3)	0.7615 (3)	0.26789 (18)	0.0556 (6)	
C1	0.8667 (3)	1.3470 (4)	0.1553 (2)	0.0464 (6)	
C2	0.9579 (4)	1.5101 (4)	0.1724 (2)	0.0590 (8)	
H2	0.9654	1.5998	0.1238	0.071*	
C3	1.0369 (4)	1.5402 (4)	0.2599 (2)	0.0625 (8)	
H3	1.0959	1.6509	0.2707	0.075*	
C4	1.0285 (3)	1.4062 (4)	0.3312 (2)	0.0539 (7)	
C5	0.9427 (3)	1.2426 (4)	0.31626 (19)	0.0472 (7)	
H5	0.9405	1.1516	0.3648	0.057*	
C6	0.8585 (3)	1.2109 (4)	0.22905 (18)	0.0422 (6)	
C7	0.7585 (3)	1.0412 (4)	0.21833 (19)	0.0430 (6)	
C8	0.6452 (3)	1.0352 (4)	0.13283 (19)	0.0529 (7)	
H8A	0.5630	1.1198	0.1430	0.063*	
H8B	0.6077	0.9029	0.1235	0.063*	
C9	0.6761 (3)	0.6340 (4)	0.3337 (2)	0.0497 (7)	
H9	0.7494	0.6483	0.3847	0.060*	
C10	0.5776 (3)	0.4669 (4)	0.33129 (17)	0.0448 (6)	
C11	0.4620 (3)	0.4417 (5)	0.25878 (18)	0.0513 (6)	
H11	0.4457	0.5356	0.2112	0.062*	
C12	0.3723 (4)	0.2815 (4)	0.2565 (2)	0.0572 (8)	
H12	0.2944	0.2689	0.2082	0.069*	
C13	0.3960 (4)	0.1366 (4)	0.3257 (2)	0.0607 (8)	
C14	0.5088 (4)	0.1620 (4)	0.3977 (2)	0.0614 (8)	
H14	0.5244	0.0681	0.4454	0.074*	
C15	0.5999 (3)	0.3245 (4)	0.4010 (2)	0.0562 (7)	
H15	0.6763	0.3382	0.4503	0.067*	
C16	0.2981 (4)	−0.0437 (6)	0.3201 (3)	0.0892 (11)	
H16A	0.3586	−0.1589	0.3278	0.134*	
H16B	0.2389	−0.0476	0.2594	0.134*	
H16C	0.2347	−0.0387	0.3695	0.134*	
C17	0.7742 (4)	1.4824 (6)	−0.0046 (3)	0.0913 (12)	
H17A	0.7455	1.6028	0.0230	0.137*	
H17B	0.7025	1.4480	−0.0572	0.137*	
H17C	0.8693	1.4984	−0.0264	0.137*	

### Atomic displacement parameters (Å^2^)

**Table:**

	*U*^11^	*U*^22^	*U*^33^	*U*^12^	*U*^13^	*U*^23^
Br1	0.0823 (2)	0.0930 (2)	0.0655 (2)	−0.0207 (2)	−0.00971 (15)	−0.02718 (19)
S1	0.0823 (6)	0.0609 (4)	0.0395 (4)	−0.0132 (4)	0.0050 (4)	−0.0079 (3)
O1	0.1165 (19)	0.0679 (16)	0.0801 (16)	−0.0059 (15)	0.0453 (14)	−0.0163 (13)
O2	0.135 (2)	0.106 (2)	0.0500 (14)	−0.0347 (17)	−0.0242 (15)	0.0036 (13)
N1	0.0768 (18)	0.0526 (13)	0.0453 (13)	−0.0107 (13)	−0.0011 (12)	0.0057 (11)
N2	0.0566 (14)	0.0422 (13)	0.0553 (14)	−0.0149 (11)	−0.0005 (11)	0.0018 (11)
N3	0.0606 (16)	0.0462 (13)	0.0585 (15)	−0.0122 (11)	0.0003 (12)	0.0013 (11)
C1	0.0480 (17)	0.0447 (14)	0.0479 (16)	−0.0029 (12)	0.0112 (13)	−0.0047 (12)
C2	0.069 (2)	0.0496 (17)	0.0604 (19)	−0.0148 (15)	0.0168 (16)	0.0001 (13)
C3	0.063 (2)	0.0513 (16)	0.074 (2)	−0.0220 (14)	0.0096 (17)	−0.0113 (16)
C4	0.0504 (16)	0.0603 (19)	0.0498 (16)	−0.0115 (14)	0.0015 (12)	−0.0193 (14)
C5	0.0506 (17)	0.0493 (15)	0.0419 (15)	−0.0055 (12)	0.0064 (13)	−0.0036 (12)
C6	0.0419 (16)	0.0429 (13)	0.0422 (14)	−0.0058 (12)	0.0077 (12)	−0.0066 (11)
C7	0.0435 (16)	0.0438 (13)	0.0420 (14)	−0.0043 (12)	0.0063 (12)	−0.0083 (12)
C8	0.0521 (18)	0.0549 (15)	0.0515 (17)	−0.0131 (13)	0.0055 (14)	−0.0046 (13)
C9	0.0485 (16)	0.0483 (15)	0.0516 (16)	−0.0026 (13)	0.0036 (13)	−0.0007 (13)
C10	0.0486 (15)	0.0410 (13)	0.0463 (14)	0.0012 (14)	0.0120 (11)	0.0020 (13)
C11	0.0622 (17)	0.0427 (13)	0.0493 (14)	−0.0018 (17)	0.0083 (13)	0.0008 (15)
C12	0.060 (2)	0.0536 (16)	0.0583 (17)	−0.0074 (15)	0.0075 (15)	−0.0110 (15)
C13	0.064 (2)	0.0451 (15)	0.076 (2)	−0.0061 (15)	0.0235 (18)	−0.0048 (15)
C14	0.067 (2)	0.0470 (16)	0.073 (2)	−0.0025 (15)	0.0199 (18)	0.0145 (15)
C15	0.0551 (19)	0.0550 (16)	0.0592 (18)	0.0025 (15)	0.0101 (14)	0.0057 (15)
C16	0.088 (2)	0.0594 (18)	0.123 (3)	−0.027 (2)	0.025 (2)	−0.007 (3)
C17	0.094 (3)	0.085 (3)	0.089 (3)	−0.005 (2)	−0.015 (2)	0.034 (2)

### Geometric parameters (Å, °)

**Table:**

Br1—C4	1.892 (3)	C8—H8A	0.9700
S1—O1	1.407 (3)	C8—H8B	0.9700
S1—O2	1.423 (3)	C9—C10	1.450 (4)
S1—N1	1.637 (3)	C9—H9	0.9300
S1—C8	1.734 (3)	C10—C15	1.384 (4)
N1—C1	1.402 (4)	C10—C11	1.392 (4)
N1—C17	1.457 (4)	C11—C12	1.364 (4)
N2—C7	1.281 (3)	C11—H11	0.9300
N2—N3	1.394 (3)	C12—C13	1.392 (4)
N3—C9	1.271 (3)	C12—H12	0.9300
C1—C2	1.394 (4)	C13—C14	1.371 (5)
C1—C6	1.408 (4)	C13—C16	1.518 (5)
C2—C3	1.374 (4)	C14—C15	1.384 (4)
C2—H2	0.9300	C14—H14	0.9300
C3—C4	1.373 (4)	C15—H15	0.9300
C3—H3	0.9300	C16—H16A	0.9600
C4—C5	1.366 (4)	C16—H16B	0.9600
C5—C6	1.392 (4)	C16—H16C	0.9600
C5—H5	0.9300	C17—H17A	0.9600
C6—C7	1.471 (4)	C17—H17B	0.9600
C7—C8	1.496 (4)	C17—H17C	0.9600
			
O1—S1—O2	119.10 (17)	S1—C8—H8B	109.5
O1—S1—N1	110.09 (15)	H8A—C8—H8B	108.1
O2—S1—N1	107.38 (15)	N3—C9—C10	121.9 (3)
O1—S1—C8	107.40 (14)	N3—C9—H9	119.1
O2—S1—C8	110.87 (15)	C10—C9—H9	119.1
N1—S1—C8	100.39 (13)	C15—C10—C11	118.3 (3)
C1—N1—C17	122.0 (3)	C15—C10—C9	119.9 (3)
C1—N1—S1	118.89 (18)	C11—C10—C9	121.7 (3)
C17—N1—S1	117.7 (2)	C12—C11—C10	121.0 (3)
C7—N2—N3	113.6 (2)	C12—C11—H11	119.5
C9—N3—N2	113.0 (2)	C10—C11—H11	119.5
C2—C1—N1	118.9 (3)	C11—C12—C13	120.7 (3)
C2—C1—C6	118.8 (3)	C11—C12—H12	119.6
N1—C1—C6	122.2 (2)	C13—C12—H12	119.6
C3—C2—C1	120.9 (3)	C14—C13—C12	118.4 (3)
C3—C2—H2	119.5	C14—C13—C16	121.7 (3)
C1—C2—H2	119.5	C12—C13—C16	119.9 (3)
C2—C3—C4	119.7 (3)	C13—C14—C15	121.3 (3)
C2—C3—H3	120.1	C13—C14—H14	119.3
C4—C3—H3	120.1	C15—C14—H14	119.3
C5—C4—C3	120.8 (3)	C14—C15—C10	120.2 (3)
C5—C4—Br1	119.2 (2)	C14—C15—H15	119.9
C3—C4—Br1	120.0 (2)	C10—C15—H15	119.9
C4—C5—C6	120.7 (3)	C13—C16—H16A	109.5
C4—C5—H5	119.7	C13—C16—H16B	109.5
C6—C5—H5	119.7	H16A—C16—H16B	109.5
C5—C6—C1	119.0 (2)	C13—C16—H16C	109.5
C5—C6—C7	118.9 (2)	H16A—C16—H16C	109.5
C1—C6—C7	122.0 (2)	H16B—C16—H16C	109.5
N2—C7—C6	118.7 (2)	N1—C17—H17A	109.5
N2—C7—C8	123.6 (2)	N1—C17—H17B	109.5
C6—C7—C8	117.7 (2)	H17A—C17—H17B	109.5
C7—C8—S1	110.75 (19)	N1—C17—H17C	109.5
C7—C8—H8A	109.5	H17A—C17—H17C	109.5
S1—C8—H8A	109.5	H17B—C17—H17C	109.5
C7—C8—H8B	109.5		
			
O1—S1—N1—C1	−64.2 (3)	N3—N2—C7—C6	177.0 (2)
O2—S1—N1—C1	164.7 (2)	N3—N2—C7—C8	−1.1 (4)
C8—S1—N1—C1	48.8 (2)	C5—C6—C7—N2	−11.9 (4)
O1—S1—N1—C17	102.5 (3)	C1—C6—C7—N2	171.1 (3)
O2—S1—N1—C17	−28.6 (3)	C5—C6—C7—C8	166.3 (2)
C8—S1—N1—C17	−144.5 (3)	C1—C6—C7—C8	−10.6 (4)
C7—N2—N3—C9	−178.8 (2)	N2—C7—C8—S1	−139.9 (2)
C17—N1—C1—C2	−6.8 (4)	C6—C7—C8—S1	41.9 (3)
S1—N1—C1—C2	159.2 (2)	O1—S1—C8—C7	58.5 (2)
C17—N1—C1—C6	170.8 (3)	O2—S1—C8—C7	−169.9 (2)
S1—N1—C1—C6	−23.1 (4)	N1—S1—C8—C7	−56.6 (2)
N1—C1—C2—C3	176.7 (3)	N2—N3—C9—C10	−179.8 (2)
C6—C1—C2—C3	−1.1 (4)	N3—C9—C10—C15	175.6 (3)
C1—C2—C3—C4	1.3 (5)	N3—C9—C10—C11	−2.7 (4)
C2—C3—C4—C5	0.2 (5)	C15—C10—C11—C12	0.3 (4)
C2—C3—C4—Br1	−178.6 (2)	C9—C10—C11—C12	178.6 (3)
C3—C4—C5—C6	−1.8 (4)	C10—C11—C12—C13	−1.4 (4)
Br1—C4—C5—C6	177.0 (2)	C11—C12—C13—C14	2.1 (4)
C4—C5—C6—C1	2.0 (4)	C11—C12—C13—C16	−178.0 (3)
C4—C5—C6—C7	−175.1 (2)	C12—C13—C14—C15	−1.7 (4)
C2—C1—C6—C5	−0.5 (4)	C16—C13—C14—C15	178.4 (3)
N1—C1—C6—C5	−178.2 (2)	C13—C14—C15—C10	0.6 (4)
C2—C1—C6—C7	176.5 (2)	C11—C10—C15—C14	0.1 (4)
N1—C1—C6—C7	−1.2 (4)	C9—C10—C15—C14	−178.2 (2)

### Hydrogen-bond geometry (Å, °)

**Table:**

*D*—H···*A*	*D*—H	H···*A*	*D*···*A*	*D*—H···*A*
C17—H17C···O1^i^	0.96	2.64	3.546 (5)	158.

Symmetry codes: (i) −*x*+2, *y*+1/2, −*z*.
